# Musashi2 Is Required for the Self-Renewal and Pluripotency of Embryonic Stem Cells

**DOI:** 10.1371/journal.pone.0034827

**Published:** 2012-04-04

**Authors:** Erin L. Wuebben, Sunil K. Mallanna, Jesse L. Cox, Angie Rizzino

**Affiliations:** Eppley Institute for Research in Cancer and Allied Diseases, University of Nebraska Medical Center, Omaha, Nebraska, United States of America; The University of Hong Kong, Hong Kong

## Abstract

Recent studies have shown that the RNA binding protein Musashi 2 (Msi2) plays important roles during development. Msi2 has also been shown to be elevated in several leukemias and its elevated expression has been linked with poorer prognosis in these cancers. Additionally, in embryonic stem cells (ESC) undergoing the early stages of differentiation, Msi2 has been shown to associate with the transcription factor Sox2, which is required for the self-renewal of ESC. These findings led us to examine the effects of Msi2 on the behavior of ESC. We determined that ESC express two isoforms of Msi2, the larger canonical isoform (isoform 1) and a shorter, splice-variant isoform (isoform 2). Using multiple shRNA lentiviral vectors, we determined that knockdown of Msi2 disrupts the self-renewal of ESC and promotes their differentiation into cells that express markers associated with mesoderm, ectoderm, and trophectoderm. Moreover, our studies indicate that the extent of differentiation and the loss of self-renewal capacity correlate with the levels to which Msi2 levels were decreased. We extended these findings by engineering ESC to inducibly express either Msi2 isoform1 or isoform 2. We determined that ectopic expression of Msi2 isoform 1, but not isoform 2, enhances the cloning efficiency of ESC. In addition, we examined how Msi2 isoform 1 and isoform 2 affect the differentiation of ESC. Interestingly, ectopic expression of either Msi2 isoform 1 or isoform 2 does not affect the pattern of differentiation induced by retinoic acid. Finally, we show that ectopic expression of either isoform 1 or isoform 2 is not sufficient to block the differentiation that results from the knockdown of both isoforms of Msi2. Thus, it appears that both isoforms of Msi2 are required for the self-renewal of ESC.

## Introduction

Sox2 and Oct4 are required during mammalian embryogenesis and play critical roles in the self-renewal and pluripotency of embryonic stem cells (ESC). Sox2 and Oct4 must be maintained within narrow limits, as an increase as small as 2-fold in the level of either transcription factor disrupts the self-renewal of ESC and triggers their differentiation [1], [2]. To help understand how small increases in Sox2 induce the differentiation of ESC, we recently performed an unbiased proteomic screen to identify nuclear proteins that associate with Sox2 [3]. We determined that Sox2 associates with >60 proteins during the early stages of differentiation. Remarkably, Sox2-associated proteins participate in a diverse range of cellular processes, including RNA processing. Included in the RNA processing group of Sox2-associated proteins are Lin28 and Musashi2 (Msi2). Lin28, which regulates let7 miRNA, is required for the self-renewal of ESC [4], but the role of Msi2 in ESC has not been examined.

Msi2 is part of a family of RNA-binding proteins that includes Musashi1 (Msi1). Msi1 and Msi2 each contain two RNA recognition motifs, and both Msi1 and Msi2 can be expressed as more than one isoform due to alternative splicing [5]. Although the roles of Musashi proteins are far from clear, Msi1 has been shown to block the translation of Numb by binding to the 3′ UTR of Numb mRNA [6]. Interestingly, knockdown of Msi2 in two leukemic cell lines led to an increase in Numb at the protein level, but it remains to be determined whether this is a direct effect of Msi2 [7].

Musashi proteins appear to play important roles during development. Msi1 and Msi2 have been shown to contribute to the development of the nervous system, where they appear to work cooperatively to promote the maintenance of neural stem cells [8]. More recently, Msi2 has been shown to influence the behavior of hematopoietic stem cells (HSC) and their progenitors. Overexpression of Msi2 in HSC in a transgenic mouse model increased the population of HSC progenitors and decreased the population of their downstream derivatives [7]. In contrast, knockdown of Msi2 by shRNA in lymphomyeloid progenitors led to an increase in the proportion of more mature differentiated myeloid cells [9]. The importance of Msi2 during hematopoiesis is also evident from the finding that Msi2 null mice exhibit significant defects in HSC. Interestingly, Msi2 null mice are smaller than their wild-type counterparts, they are produced at lower than expected frequencies, and Msi2 null mice are infertile when mated together [10], [11]. The reasons for each of these defects remain to be determined.

In addition to influencing development, Msi2 has also been linked to tumorigenesis. Importantly, recent studies have shown Msi2 is overexpressed in chronic myelogenous leukemia (CML) and acute myeloid leukemia (AML) [7], [10]. In CML, Msi2 is elevated ∼10-fold during the more aggressive blast crisis phase than in the chronic phase, and elevated Msi2 expression in CML has been linked to relapse and poorer prognosis [10]. Consistent with these findings, knockdown of Msi2 by shRNA in blast crisis CML cells led to a more differentiated cell population and diminished proliferation of the diseased cells [10]. Interestingly, knockdown of Msi2 in several leukemic cell lines reduced their proliferation and led to increased differentiation and apoptosis [7].

Given the contribution of Msi2 to tumorigenicity, its roles during development and its association with Sox2 in ESC, we explored the possibility that altering the expression of Msi2 influences the self-renewal and pluripotency of ESC.

## Results

### ESC express and require Msi2

**Figure 1:**
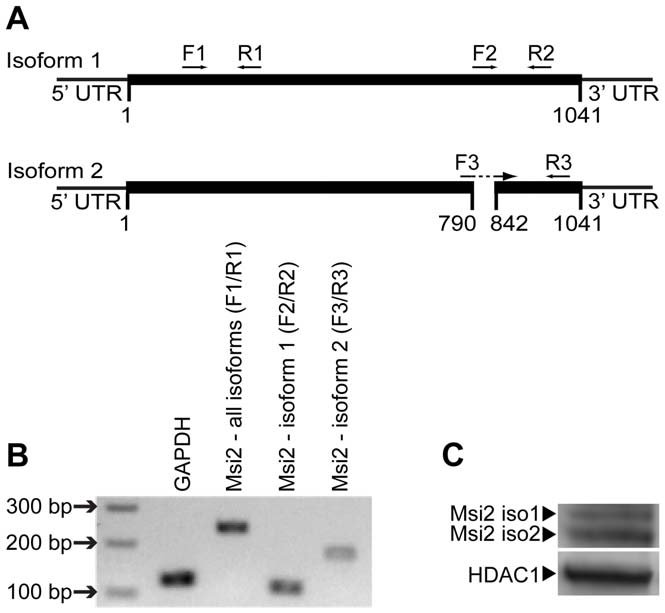
Expression of Msi2 mRNA and protein in ESC. (**A**) Primers for RT-qPCR were designed to be specific for both isoforms of Msi2, isoform 1 or isoform 2. Primer set F1/R1 amplifies sequences present in both isoform 1 and 2. Primer sets F2/R2 and F3/R3 are specific to isoform 1 and isoform 2, respectively. (**B**) DNA fragments generated via RT-qPCR using RNA isolated from D3 ESC and the primer sets indicated were separated over a 2% agarose gel. (**C**) Western blot analysis of Msi2 isoforms expressed in nuclear extracts isolated from D3 ESC. Isoform 2 runs slightly faster than isoform 1 (35.7 kDa and 36.9 kDa, respectively) [5]. HDAC1 protein was used as a loading control.

To study the role of Msi2 in mouse ESC, we initially examined whether ESC express more than one isoform of Msi2. Studies in other systems [5], [12] identified two isoforms of Msi2: isoform 1 (full length) consisting of 346 amino acids and isoform 2 consisting of 328 amino acids (Fig. 1A). Using primers specific to Msi2 isoform 1 and isoform 2, we determined that D3 ESC express both isoforms of Msi2 at the RNA level (Fig. 1B). In addition, we determined by western blot analysis that ESC express both isoforms of Msi2 at the protein level (Fig. 1C).

**Figure 2:**
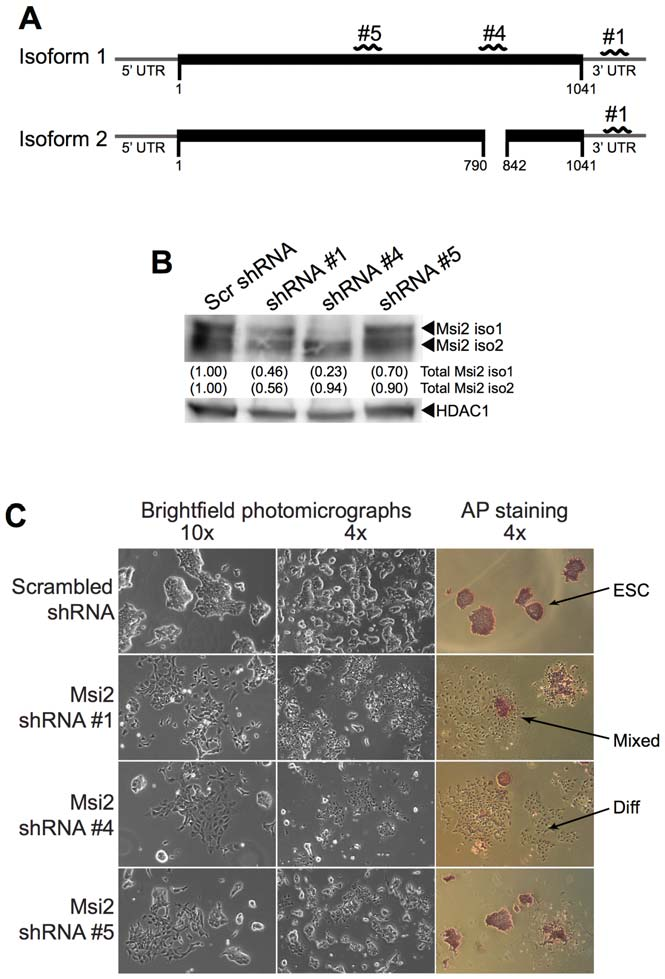
Knockdown of Msi2 results in the differentiation of ESC. (**A**) Regions of Msi2 mRNA targeted by shRNA #1, shRNA #4, and shRNA #5. (**B**) The D3 ESC were infected with lentiviruses that express scrambled (Scr) shRNA, shRNA #1, shRNA #4, or shRNA #5 sequences. Two days after infection, the cells were subjected to puromycin selection for 24 hours. After selection, the cells were subcultured and grown for an additional 24 hours before nuclear extracts were harvested for western blot analysis. HDAC1 was used as the loading control for quantification. (**C**) Bright field photomicrographs of cells subcultured at 5,000 cells per cm^2^ were taken 7 days post-infection with each shRNA (left columns). Cells were stained with alkaline phosphatase (right column) 10 days post-infection after being subcultured at 200 cells per cm^2^. Arrows point to colonies that exhibit a morphology characteristic of ESC (ESC), a morphology consisting of ESC and differentiated cells (Mixed), or a morphology characteristic of differentiated cells (Diff).

To investigate the role of Msi2 in ESC, we examined how ESC would be affected by the knockdown of Msi2. For this purpose, we used lentiviral vectors that express shRNA directed at both isoforms (shRNA #1 and shRNA #5) and only isoform 1 (shRNA #4) (Fig. 2A). Specifically targeting isoform 2 will be more difficult, as isoform 2 shares the entirety of its sequence with isoform 1, differing only through an omission of a small region. Thus, to target isoform 2, an shRNA sequence will need to bridge the region that is spliced out from isoform 1. As a control, we also infected ESC with a lentivirus that expresses a scrambled shRNA sequence, which we previously determined does not influence the behavior of D3 ESC [13]. Initially, we examined the knockdown of Msi2 by comparing the protein levels of Msi2 in ESC infected with the lentiviral vector that expresses the scrambled shRNA control with the levels of Msi2 in cells infected with lentiviral vectors that express shRNA #1, #4 or #5. We determined that infection of ESC with the lentiviral vector that expresses shRNA #1 caused a significant reduction in both isoforms of Msi2, and infection of ESC with the lentiviral vector that expresses #4 caused a significant reduction in Msi2 isoform 1, but not isoform 2 (Fig. 2B). In contrast, the lentiviral vector that expresses shRNA #5 appears to induce only a modest reduction in isoform 1 (∼30%) and small reduction in isoform 2 (∼10%).

Examination of the infected cells by light microscopy indicated that ESC infected with the control lentiviral vector, which expresses the scrambled shRNA sequence, did not induce morphological changes in the cells. In contrast, lentiviral vectors #1 and #4 caused extensive morphological differentiation (Fig. 2C). As expected from the effects on Msi2 protein expression (Fig. 2B), lentiviral vector #5 caused significantly less differentiation. To further characterize the observed change in morphology, we stained cells infected with the various shRNA constructs with alkaline phosphatase (AP), a cell-surface marker associated with pluripotency. Reduced AP-staining intensity in ESC infected with Msi2 shRNA constructs #1 and #4 corroborated our observation that knockdown of Msi2 impairs the ability of ESC to self-renew (Fig. 2C). To determine whether the differentiation observed was due to a loss in essential pluripotency factors, we conducted western blot analysis to examine the levels of Sox2 and Oct4 following the knockdown of Msi2. Interestingly, the levels of Sox2 and Oct4 were not significantly altered after the knockdown of Msi2 (Fig. S1).

**Figure 3:**
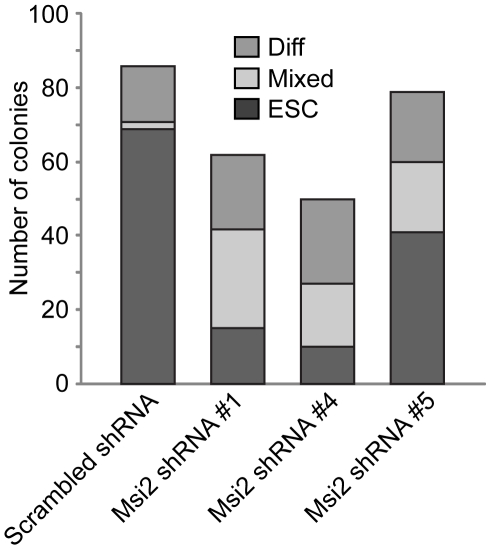
Knockdown of Msi2 decreases the cloning efficiency of ESC. D3 ESC infected with lentiviral constructs that express Scr, Msi2#1, Msi2#4, or Msi2#5 shRNA sequences were subcultured at 200 cells per cm^2^. Six days later an observer unaware of sample designation scored colonies as ESC, Mixed or differentiated (Diff).

To quantitate the extent of differentiation induced by the knockdown of Msi2, virally infected cells were plated at clonal density. Six days later, colonies were scored by an observer unaware of sample designation as ES cell colonies, differentiated colonies or mixed colonies consisting of both ESC and differentiated cells. Typical of unmodified ESC, a high percentage of D3 ESC expressing the scrambled shRNA formed ES cell colonies (∼80%) and relatively few mixed and differentiated colonies (Fig. 3). In contrast, D3 ESC infected with lentiviral vectors expressing either shRNA #1 or shRNA #4 formed far fewer ES cell colonies (<15% in the case of shRNA #4) and a large percentage of mixed and differentiated cell colonies. In addition, shRNA #5 only modestly reduced the number of ES cell colonies (by ∼40%) and increased the number of mixed and differentiated colonies. Importantly, our studies show that the extent of differentiation and the loss of self-renewal capacity correlate with the level to which Msi2 levels were decreased. Equally importantly, our studies indicate that the knockdown of isoform 1 induces the differentiation of ESC. Studies discussed below suggest that the self-renewal of ESC may also require expression of Msi2 isoform 2.

### Msi2 knockdown alters gene expression

To further characterize the differentiation of ESC following the knockdown of Msi2, we initially examined the changes in gene expression by microarray analysis. Changes in global RNA expression were determined by comparing the RNA expression profiles of ESC that express either the scrambled shRNA or shRNA #1 by microarray. Of 29,000 transcripts examined, we determined that cells infected with Msi2 shRNA #1 exhibited increased expression of 40 genes ≥2.5-fold (Table 1) and decreased expression of 29 genes ≥2.5-fold (Table 2) compared to cells infected with scrambled shRNA. Broadening our parameters to genes whose expression increased or decreased by ≥2-fold (Fig. S2A, Tables S1 and S2) expanded these subsets with an additional 49 and 46 genes, respectively. Gene ontology analysis (Fig. S2B, Tables S3 and S4) indicated that a large percentage of the genes that exhibited >2-fold increased gene expression play roles in cell signaling (e.g. Tspan2, Irs2, Sfrp2, Ctgf) and cellular structure (e.g. Acta2, Actc1, Cald1, Myl9); whereas, genes that exhibited >2-fold decrease in expression participate in development (e.g. Amot, Pdgfr, Lama1) and metabolism (e.g. Nostrin, Nrg1, Inhbb). Given the morphological changes that accompany differentiation, in particular the increase in cytoplasmic to nuclear ratio, increases in genes associated with cellular structure is not surprising. Similarly, the large change in the expression of genes associated with metabolic processes when Msi2 is knocked down parallels the large changes in metabolic gene expression when somatic cells are reprogrammed to induced pluripotent stem cells [14]. A complete list of the differentially expressed genes and their associated ontologies are provided in a heatmap (Tables S3 and S4).

**Table 1:** Genes more highly expressed (>2.5-fold) in Msi2-shRNA #1 treated ESC.

Gene Description	Gene Symbol	Gene Accession	Msi/Scr
actin, alpha 2, smooth muscle, aorta	Acta2	NM_007392	17.8741
fibronectin type III domain containing 3C1	Fndc3c1	NM_001007580	10.4233
cytochrome P450, family 2, subfamily b, polypeptide 23	Cyp2b23	NM_001081148	9.95679
guanylate binding protein 2	Gbp2	NM_010260	9.71739
myosin, light polypeptide 9, regulatory	Myl9	NM_172118	9.12257
transgelin	Tagln	NM_011526	8.76221
protogenin homolog (Gallus gallus)	Prtg	NM_175485	5.76868
calponin 1	Cnn1	NM_009922	5.19768
sema domain, immunoglobulin domain (Ig), short basic domain, secreted, (semaphorin) 3E	Sema3e	NM_011348	5.10546
fermitin family homolog 1 (Drosophila)	Fermt1	NM_198029	4.58914
G protein-coupled receptor 177	Gpr177	NM_026582	4.57058
lymphoid enhancer binding factor 1	Lef1	NM_010703	4.29654
lysophosphatidic acid receptor 4	Lpar4	NM_175271	4.24447
tropomyosin 1, alpha	Tpm1	NM_024427	4.20367
collectin sub-family member 12	Colec12	NM_130449	4.11384
von Willebrand factor A domain containing 5A	Vwa5a	NM_172767	4.00526
cadherin 2	Cdh2	NM_007664	3.83405
inhibitor of DNA binding 2	Id2	NM_010496	3.65226
peripheral myelin protein 22	Pmp22	NM_008885	3.64122
latrophilin 2	Lphn2	NM_001081298	3.49388
sema domain, transmembrane domain (TM), and cytoplasmic domain, (semaphorin) 6A	Sema6a	NM_018744	3.46536
latrophilin 2	Lphn2	NM_001081298	3.26815
forkhead box I3	Foxi3	NM_001101464	3.26699
brachyury	T	NM_009309	3.25879
calponin 2	Cnn2	NM_007725	3.24541
Meis homeobox 2	Meis2	NM_001136072	3.22486
latrophilin 2	Lphn2	NM_001081298	3.13186
actin, alpha, cardiac muscle 1	Actc1	NM_009608	3.11953
latrophilin 2	Lphn2	NM_001081298	3.00825
latrophilin 2	Lphn2	NM_001081298	2.94293
thrombospondin 1	Thbs1	NM_011580	2.80869
Ras-related GTP binding D	Rragd	NM_027491	2.76052
matrix metallopeptidase 25	Mmp25	NM_001033339	2.71228
secreted frizzled-related protein 2	Sfrp2	NM_009144	2.70216
tetraspanin 7	Tspan7	NM_019634	2.68492
paired box gene 6	Pax6	NM_013627	2.62556
double homeobox B-like	Duxbl	NM_183389	2.59041
double homeobox B-like	Duxbl	NM_183389	2.59041
transmembrane protein 47	Tmem47	NM_175771	2.54062
carbonic anhydrase 4	Car4	NM_007607	2.5016

**Table 2:** Genes more highly expressed (>2.5-fold) in control scr-shRNA treated ESC.

Name	Gene Symbol	Gene Accession	Msi/Scr
cubilin (intrinsic factor-cobalamin receptor)	Cubn	NM_001081084	0.17396
nuclear RNA export factor 7	Nxf7	NM_130888	0.18541
nitric oxide synthase trafficker	Nostrin	NM_181547	0.20747
apolipoprotein B mRNA editing enzyme, catalytic polypeptide 2	Apobec2	NM_009694	0.27086
serglycin	Srgn	NM_011157	0.27474
carcinoembryonic antigen-related cell adhesion molecule 1	Ceacam1	NM_001039185	0.28154
KDEL (Lys-Asp-Glu-Leu) endoplasmic reticulum protein retention receptor 3	Kdelr3	NM_134090	0.28702
transcription factor EC	Tcfec	NM_031198	0.29192
klotho beta	Klb	NM_031180	0.29292
solute carrier family 44, member 3	Slc44a3	NM_145394	0.29672
laminin, alpha 1	Lama1	NM_008480	0.30992
forkhead box Q1	Foxq1	NM_008239	0.31102
GLI pathogenesis-related 1 (glioma)	Glipr1	NM_028608	0.32064
HNF1 homeobox B	Hnf1b	NM_009330	0.32633
dickkopf homolog 1 (Xenopus laevis)	Dkk1	NM_010051	0.3305
disabled homolog 2 (Drosophila)	Dab2	NM_023118	0.33297
SRY-box containing gene 7	Sox7	NM_011446	0.34529
fatty acid binding protein 3, muscle and heart	Fabp3	NM_010174	0.34669
inhibin beta-B	Inhbb	NM_008381	0.35116
fatty acid binding protein 3, muscle and heart	Fabp3	NM_010174	0.3513
N-acetylneuraminate pyruvate lyase	Npl	NM_028749	0.35156
stearoyl-Coenzyme A desaturase 1	Scd1	NM_009127	0.36289
ankyrin repeat domain 33B	Ankrd33b	NM_027496	0.36428
legumain	Lgmn	NM_011175	0.37277
epithelial membrane protein 1	Emp1	NM_010128	0.37382
GATA binding protein 4	Gata4	NM_008092	0.38549
pterin 4 alpha carbinolamine dehydratase/dimerization cofactor of hepatocyte nuclear factor 1 alpha (TCF1) 1	Pcbd1	NM_025273	0.39076
serine peptidase inhibitor, Kazal type 3	Spink3	NM_009258	0.39558
DNA-damage regulated autophagy modulator 1	Dram1	NM_027878	0.39648

**Figure 4:**
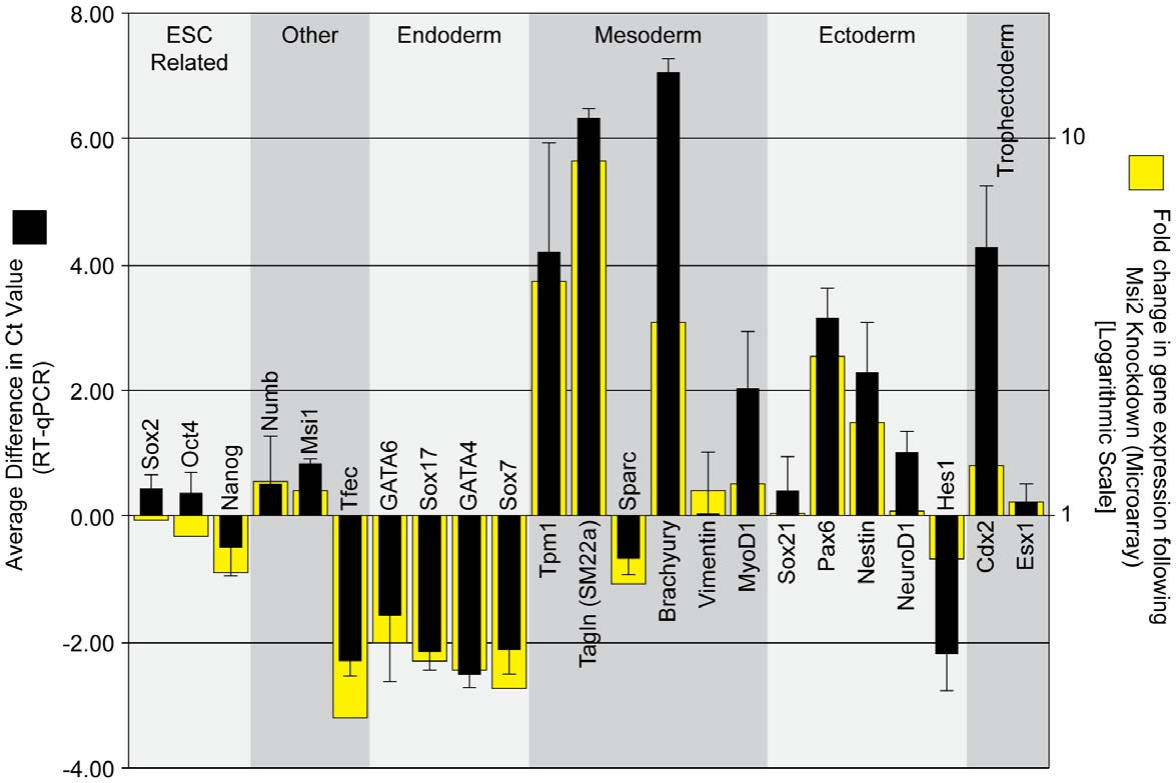
Knockdown of Msi2 in ESC leads to the expression of lineage-specific markers. Seven days post-infection, RNA was isolated from the D3 ESC infected with lentiviruses that express either the scrambled (Scr) shRNA sequence or the Msi2 shRNA #1 sequence. Microarray analysis was used to assess global changes in gene expression (Fig. S2A). RNA expression for a select set of genes (yellow bars) is indicated as fold change, where a value >1 represents an increase in expression in cells treated with shRNA #1. All microarray data is available on Gene Expression Omnibus (Accession No. GSE33882, GEO, http://www.ncbi.nlm.nih.gov/geo/). Expression of this gene subset was verified by RT-qPCR (black bars). Threshold cycle (Ct) values were calculated by normalizing all Ct values to GAPDH then subtracting the Ct value for cells infected with Msi2 shRNA #1 from the Ct value for cells infected with the scrambled shRNA lentivirus. A positive Ct value indicates an increase in the level of the transcript in the Msi2 knockdown cells. Multiple rounds of RT-qPCR were used to calculate an average change in Ct value, and error bars represent standard deviation.

To validate our microarray analysis, 23 genes were examined more closely using RT-qPCR, which is a more quantitative approach to examine transcript expression. For this analysis, we examined a number of genes that increased or decreased according to our microarray data, and a number of genes critical for maintaining pluripotency in ESC. In concordance with our microarray data, analysis by RT-qPCR indicates that a number of genes associated with mesoderm development (Tpm1, Tagln, Brachyury, MyoD1), ectoderm development (Pax6, Nestin) and trophectoderm development (Cdx2, Esx1) were elevated when Msi2 was knocked down (Fig. 4). Additionally, a number of markers associated with endoderm development (Gata6, Sox17, Gata4, Sox7) were reduced as determined by both our microarray and RT-qPCR (Fig. 4). We also examined the expression of Numb and Msi1 mRNA by RT-qPCR, both of which exhibited a small increase when Msi2 was knocked down. The small increase in Msi1 mRNA may reflect a compensatory mechanism that coordinates the expression of Msi1 and Msi2.

### Msi2 enhances the cloning efficiency of ESC

Because of our observation that the knockdown of Msi2 leads to the loss of self-renewal in ESC, we examined whether the elevation of Msi2 would enhance the self-renewal of ESC. Recent studies have shown that elevating Msi2 helps in the maintenance of hematopoietic and tumor stem cells [7], [9], [10]. However, it was not clear whether Msi2, in particular isoform 1 or isoform 2 could enhance ESC self-renewal. To examine this possibility, we engineered ESC for doxycycline (Dox)-inducible overexpression of Flag-tagged Msi2, as described in the Methods section. Briefly, D3 ESC were infected with a lentivirus that constitutively expresses the reverse tet transactivator (rtTA), which binds Dox to mediate inducible transgene expression. These cells were then infected with a second lentivirus that expresses either Flag-tagged Msi2 isoform 1 (i-Msi2.1-D3 ESC) or isoform 2 (i-Msi2.2-D3 ESC) when the cells are exposed to Dox.

**Figure 5:**
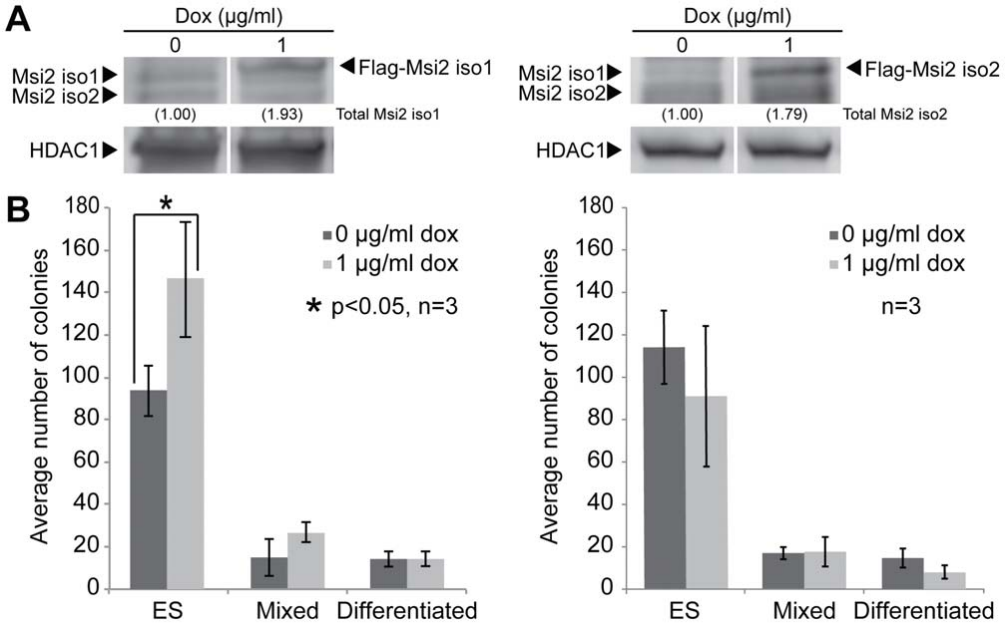
Overexpression of Msi2 in ESC increases the cloning efficiency of ESC. (**A**) Western blot analysis of Msi2 present in D3 ESC stably infected with a lentivirus for the inducible expression of Flag-tagged Msi2 isoform 1 (left) or Flag-tagged Msi2 isoform 2 (right) in the presence of 1 µg/ml Dox. (**B**) Effects of inducing Flag-tagged Msi2 isoform 1 (left) or Flag-tagged Msi2 isoform 2 (right) on the cloning efficiency of D3 ESC. The cells were plated at clonal density (200 cells per cm^2^) and exposed to 1 µg/ml Dox for 5 days. The error bars are standard error of the mean. This experiment was repeated twice and similar results were obtained.

Using this system, we determined that addition of Dox leads to a small increase (∼1.8 fold) in Flag-Msi2-isoform 1 (Fig. 5A, left) and increases the cloning efficiency of ESC, specifically the number of ES cell colonies that form (Fig. 5B, left). Conversely, ∼2-fold overexpression of Flag-Msi2-isoform 2 (Fig. 5A, right) in i-Msi2.2-D3 ESC had no significant effect on cloning efficiency, in particular the number of ES cell colonies that formed (Fig. 5B, right). As a control, D3 ESC engineered for the inducible expression of luciferase did not demonstrate significant changes in the distribution of colony morphologies upon the addition of Dox (data not shown).

### Msi2 isoform 1 or Msi2 isoform 2 on their own do not rescue the knockdown of both isoforms of Msi2

Our initial experiment in which shRNA #4 was used to target only isoform 1 suggested that Msi2 isoform 2 may not be required to support the self-renewal of ESC (Fig. 2). This finding, coupled with the observation that Msi2 isoform 1 enhances the self-renewal of ESC, led us to examine whether isoform 1 alone is sufficient to support the self-renewal of ESC.

**Figure 6:**
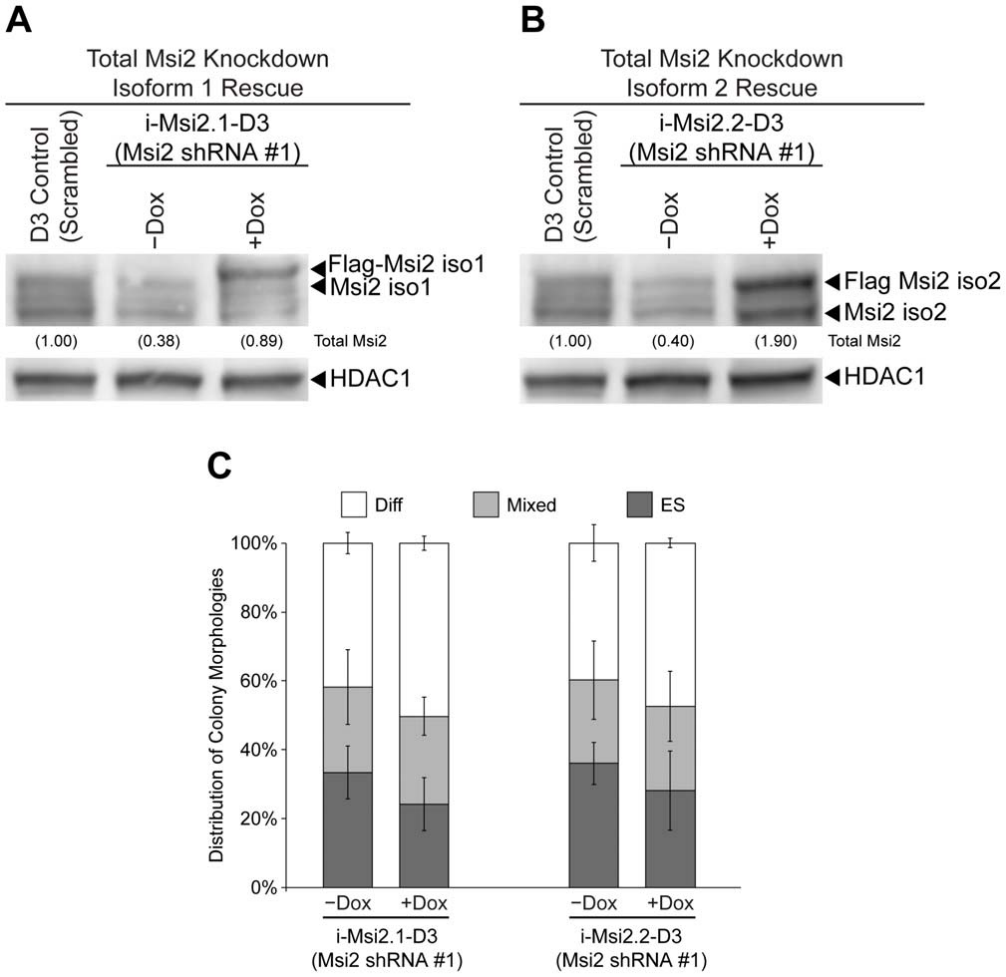
Rescue of Msi2 knockdown using an epitope-tagged overexpression construct. Cells engineered to overexpress Flag-tagged Msi2 isoform 1 or Flag-tagged Msi2 isoform 2, i-Msi2.1-D3 and i-Msi2.2-D3 respectively, were plated at 10,500 cells/cm^2^ in the presence or absence of 2 µg/ml Dox. Cells were infected with lentiviral constructs that express Msi2 shRNA #1 for 48 hours. One day later, the cells were subcultured at 600 cells/cm^2^ in the continued presence or absence of 2 µg/ml Dox and the remaining cells harvested for protein extracts. Western blot analysis was used to monitor the levels of Msi2 in i-Msi2.1-D3 (**A**) or i-Msi2.2-D3 (**B**) stably infected with shRNA #1 lentivirus in the presence or absence of 2 µg/ml Dox. D3 ESC infected with the lentiviral vector that expresses the scrambled shRNA was used as a control. (**C**) Effects of inducing Flag-tagged Msi2 isoform 1 (left) or Flag-tagged Msi2 isoform 2 (right) on the cloning efficiency of D3 ESC following Msi2 knockdown. Six days following subculture, two observers unaware of sample designation scored colonies as ESC, Mixed or differentiated (Diff). The error bars are standard deviation between the average percentages as scored by the two observers. This experiment was repeated twice and similar results were obtained.

To determine whether Msi2 isoform 1 is sufficient to support ESC self-renewal, i-Msi2.1-D3 ESC and i-Msi2.2-D3 ESC were cultured in the absence or presence of Dox (2 µg/mL) for 24 hours. Next, the cells cultured in the presence or absence of Dox were infected with Msi2 shRNA #1 lentivirus, which targets both isoforms of Msi2. Cells pretreated with Dox were cultured in the presence of Dox for the entire experiment. Western blot analysis of proteins isolated from i-Msi2.1-D3 ESC verified that total Msi2 levels were reduced (∼60%) in cells cultured in the absence of Dox; whereas, total Msi2 levels were near normal (∼90%) when the infected cells were maintained in the presence of Dox, due in part to exogenous expression from the transgene (Fig. 6A). Additionally, in i-Msi2.2-D3 ESC infected with shRNA #1, Msi2 levels were knocked down (∼60%, relative to endogenous Msi2) in the minus Dox control, but elevated ∼2-fold when cultured in the presence of Dox (Fig. 6B). The reason for the intensely staining band observed at the level of Msi2 isoform 2 in i-Msi2.2-D3 ESC cultured in the presence of Dox is not completely clear. We suspect that this is due to alternative translation start from our exogenous Flag-Msi2 isoform 2 transcript, which retains the endogenous Msi2 start codon.

To quantify any changes in self-renewal efficiency, i-Msi2.1-D3 ESC and i-Msi2.2-D3 ESC expressing their respective isoforms of Msi2 and infected with Msi2 shRNA #1 were subcultured 72 hours after infection with shRNA lentivirus, and plated at clonal density. Our findings indicate that elevation of Msi2 isoform 2 did not block the differentiation of ESC following the knockdown of both isoforms of Msi2 (Fig. 6C). Interestingly, isoform 1 was also unable to block the differentiation of ESC following the knockdown of Msi2 (Fig. 6C). Thus, our data suggests that the expression of isoforms 1 and 2 are both necessary to support the self-renewal of ESC.

### Elevation of Msi2 during differentiation does not bias differentiation

**Figure 7:**
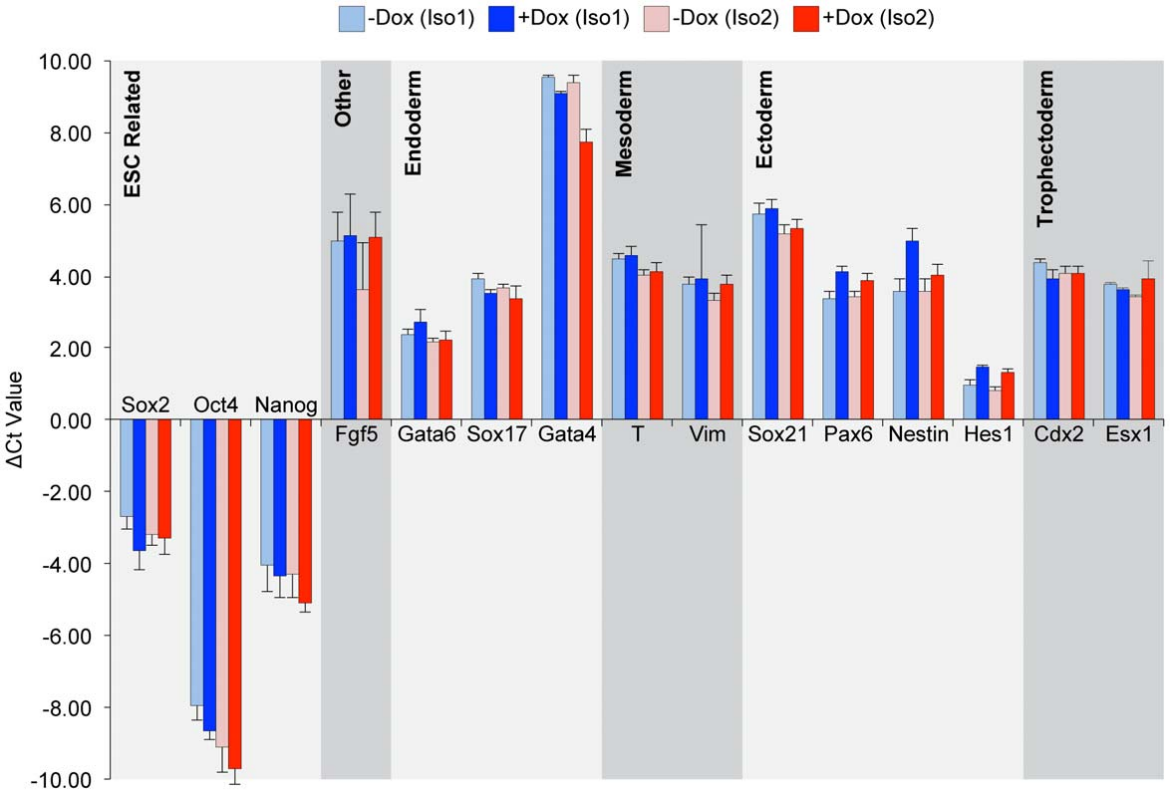
Effects of Msi2 overexpression on the differentiation of ESC. i-Msi2.1-D3 and i-Msi2.2-D3 were treated with or without 1 µg/ml Dox for 48 hours prior to plating at 4,500 cells/cm^2^. Cells were treated with and without Dox in the presence of 5 µM RA for 48 hours, followed by the removal of LIF for 48 hours to further the differentiation of the cells. RNA was isolated from these cells and the expression of a subset of differentially expressed genes was verified by RT-qPCR, as described in the Materials and Methods. Threshold cycle (Ct) values were normalized to GAPDH and represent the difference in transcript levels between the indicated treatment condition and undifferentiatied ESC. A positive Ct value indicates an increase in the level of the transcript in the differentiated cells, as indicated. Multiple rounds of RT-qPCR were used to calculate an average change in Ct value, and error bars represent standard deviation.

Because of the role of Msi2 in hematopoietic stem cell maintenance and neural development, we examined whether induction of Msi2 could skew the differentiation of ESC toward specific developmental lineages. For this purpose, i-Msi2.1-D3 and i-Msi2.2-D3 ESC, expressing their respective forms of Msi2, were differentiated using retinoic acid (RA). More specifically, i-Msi2.1-D3 and i-Msi2.2-D3 ESC were cultured continuously in the absence or presence of Dox. Two days after the addition of Dox to cells, RA was added to the culture medium, and cells were allowed to grow an additional 4 days. After treatment of cells with RA for 4 days (with and without Dox), RNA was isolated and examined by RT-qPCR analysis as described in the Methods. As expected, the pattern of differentiation induced by RA in the absence of Dox was highly similar for i-Msi2.1-D3 and i-Msi2.2-D3 ESC. More importantly, treatment of these ESC with Dox, which induces ectopic expression of Msi2 isoform 1 and Msi2 isoform 2, respectively (Fig. 5 and 6), did not alter the pattern of RA-induced differentiation (Fig. 7).

## Discussion

In this study, we demonstrate that ESC express two isoforms of Msi2, and we determined that the knockdown of Msi2 disrupts the self-renewal of ESC and induces their differentiation. Moreover, our studies demonstrate that the extent of differentiation and the loss of self-renewal capacity correlates with the extent to which Msi2 levels were decreased. Remarkably, the knockdown of Msi2 causes ESC to differentiate despite continued expression of both Sox2 and Oct4. Similar results were observed in a related study, in which barrier to autointegration factor 1 (Banf1) was knocked down in human ESC [13]. In that report, human ESC lost their capacity for self-renewal following Banf1 knockdown even though the localization and nuclear expression of Sox2 and Oct4 did not change. Furthermore, we have recently demonstrated that elevating Oct4, Sox2, Klf4 and c-Myc each ∼2- to 3-fold does not disrupt the self-renewal and pluripotency of ESC; whereas, simultaneously elevating only Oct4 and Sox2 to a similar extent induces the differentiation of ESC [15]. Thus, it appears that ESC are not dependent on the absolute levels of Sox2 and Oct4, but rather on the levels of these two critical transcription factors relative to other essential proteins. Together our findings, including those reported here for Msi2, suggest that the knockdown of Msi2 may alter the critical balance of Sox2 and Oct4 relative to other essential proteins required for the self-renewal and pluripotency of ESC.

We also determined that overexpression of Msi2 isoform 1, but not isoform 2, enhances the cloning efficiency of ESC, which is a measure of their self-renewal capacity. However, we determined that ectopic expression of either Msi2 isoform 1 or isoform 2 does not block the differentiation of ESC when both isoforms of Msi2 are knocked down. Moreover, ectopic expression of Msi2 isoform 1 or isoform 2 does not appear to alter the pattern of differentiation induced by the treatment of ESC with RA. Thus, our findings suggest that both isoforms of Msi2 are required to maintain the self-renewal of ESC.

Although knockdown of Msi2 isoform 1 is sufficient to induce the differentiation of ESC (Fig. 2 shRNA #4), a more rigorous demonstration that ESC strictly require Msi2 isoform 2 will require considerably more work. The most direct method for addressing this question would be to selectively knockdown Msi2 isoform 2. As mentioned earlier, addressing this question will require an shRNA that only targets isoform 2, which is currently not available. Thus far, this question has not been addressed in any study, including the recent reports that demonstrated prominent roles of Msi2 in the function HSC, CML and AML [7], [9], [10]. In these seminal studies, the shRNA sequences used targeted both isoforms of Msi2.

Our studies raise another important question. How does Msi2 regulate the behavior of ESC? Previous studies demonstrate that Msi1 binds to target mRNA transcripts to prevent their association with the ribosome and other translation machinery [6]. If Msi2 functions through a similar mechanism to block the translation of several critical RNAs, it will be important to determine which RNAs are targeted. Moreover, it would be interesting to compare which transcripts Msi2 targets in different cellular contexts, including ESC, HSC and tumor cells. Finally, it will be important to determine whether Msi2 plays a role during embryogenesis. Although our studies argue that ESC require Msi2, gene ablation studies argue that Msi2 is not absolutely required for embryogenesis [10], [11]. However, it remains to be determined whether the reduced frequency of null Msi2 mice is the result of minor defects during embryogenesis [10], [11]. Moreover, it is possible that Msi1 can compensate for the absence of Msi2 during embryogenesis, but is unable to for ESC grown in culture.

## Materials and Methods

### Cell culture

D3 ESC were obtained from T. Doetschman [16]. Stock cultures of D3 mouse ESC and their genetically modified derivatives (see below) were cultured as described previously [2]. Culture medium was supplemented with 5 ng/ml leukemia inhibitory factor (LIF). To differentiate ESC, the cells were treated with 5 µM retinoic acid (RA) for 4 days.

### ESC that inducibly express i-Msi2.1 and i-Msi2.2

To produce ESC that express either Flag-tagged Msi2 isoform 1 or Flag-tagged Msi2 isoform 2, D3 ESC were first infected with the lentiviral vector pLVX-TetOn-Advanced (#632162 Clontech, Mountain View, CA), which we modified by replacing the CMV promoter with a PGK promoter. (We refer to this modified vector as pLVX-PGK-TetOn-Advanced.) We isolated virally infected cells, referred to as D3-rtTA ESC, after treatment with 300 µg/mL G418 sulfate (#631308, Clontech) for 6 days. To produce i-Msi2.1-D3 ESC and i-Msi2.2-D3 ESC, D3-rtTA ESC were infected with the virus pLVX-Tight-Puro (Clontech), which was engineered to express either Flag-tagged Msi2 isoform 1 or Flag-tagged Msi2 isoform 2, respectively. Twenty-four hours after infection, D3-rtTA ESC were cultured in the presence of 5 µg/ml puromycin for 48 hours to select for infected cells.

### Construction of Lentiviral vectors

The CMV promoter responsible for driving the expression of the neomycin resistance gene in pLVX-Tet-On-Advanced (Clontech, Mountain View, CA, #632162) was replaced with a PGK promoter. For this purpose, the PGK promoter from pLVX-Tight-Puro (Clontech, #632162) was amplified by PCR, with primers that introduce ClaI and BamH1 restriction sites, upstream and downstream of the promoter, respectively. The sequence of the upstream primer for amplifying the PGK promoter was: CAGTTT**ATCGAT**TACCGGGTAGGGGAGGCGCTTTTCCCAAGGCAGTCTGG (ClaI site in bold font), and the sequence for the downstream primer was: CATGGT**GGATCC**CGAAAGGCCCGGAGATGAGGAAGAGGAGAACAGCGCGG (BamHI site in bold font). The PGK PCR product was digested with ClaI and BamH1 restriction enzymes, and the fragment was ligated into pLVX-Tet-On-Advanced, previously treated with ClaI and BamHI to remove the CMV promoter. The resulting plasmid produced pLVX-PGK-TetOn-Advanced.

To produce viruses for inducible expression of Flag-tagged Msi2 isoform 1 and Flag-tagged Msi2 isoform 2, we first cloned the coding sequence for Msi2 isoform1 and the coding sequence for Msi2-isoform2 from RNA isolated from D3 ESC. After cDNA synthesis [2], the Msi2 coding sequences were amplified by PCR. For this purpose, primers were designed to add a BamHI restriction enzyme site, Kozak sequence, and a Flag peptide to the N-terminus of the Msi2 coding sequence, as well as 3 stop codons and an EcoRI restriction enzyme site to the C-terminus. The upstream PCR primer used was: ATCGC**GGATCC**GCCACCATGGACTACAAGGACGACGATGACAAGATGGAGGCAAATGGGAGCCCA (BamHI restriction enzyme site is shown in bold font, followed by the Kozak sequence, then the Flag peptide sequence, which is underlined). The downstream primer used was: TACCG**GAATTC** *TTATTATCA*GTGGTATCCATTTGTAAAGGCCGTTGC (EcoRI restriction enzyme site in bold font and stop codons in italicized font). The Flag-Msi2 products were digested with BamH1 and EcoRI restriction enzymes, and the fragments were ligated into pBluescript II KS+ (Stratagene), previously digested with BamH1 and EcoRI in the multiple cloning site. Due to the design of the primers, both Msi2 isoforms 1 and 2 were amplified via PCR, and ligated into pBluescript II KS+. Sequencing of this library was performed by UNMC High-Throughput DNA Sequencing Core to identify Flag-Msi2 isoform 1 or Flag-Msi2 isoform 2 clones. Once identified, Flag-Msi2 isoform 1 or Flag-Msi2 isoform 2 fragments were isolated from the pBluescript II KS+ plasmids by digestion with BamH1 and EcoRI, and ligated into pLVX-Tight-Puro previously digested with the same enzymes. Lentiviral particles were produced from pLVX-Tight-Puro-Flag-Msi2 isoform 1, pLVX-Tight-Puro-Flag-Msi2 isoform 2, or pLVX-Tight-Puro-Luc (632162, Clontech) in 293T cells as described previously [13].

### Lentiviral production

Production of lentiviruses in 293T cells, including the lentivirus that expresses the scrambled shRNA sequence, has been described previously [13]. Lentiviral vectors for expression of shRNA sequences that target mouse Msi2 were obtained from Open Biosystems (RMM4534-NM_054043, Huntsville, AL). Msi2 shRNA lentiviral constructs #1, #4, and #5 used in this study correspond to TRCN0000071973, TRCN0000071976, and TRCN0000071977 (Open Biosystems), respectively. Sequences of these shRNAs are provided in Table 3.

**Table 3:** Oligonucleotides.

Msi2 shRNA	Clone ID	Mature sense sequence
Msi2 shRNA #1	TRCN0000071973	CCCAGCTTAATATCTAGTTAA
Msi2 shRNA #4	TRCN0000071976	GCTACAGTGCTCAACCGAATT
Msi2 shRNA #5	TRCN0000071977	CCACCATGAGTTAGATTCCAA

Primer Set	Primer name	RT-qPCR Primer Sequence (5′⇒3′)	Amplicon size
Msi2 F1/R1	Msi2-F1	CAACGACTCCCAGCACGAC	237 bp
	Msi2-R1	GTCAATCGTCTTGGAATCTAACTC	
Msi2 F2/R2	Msi2-iso1-F	GTGCTCAACCGAATTTTGGC	106 bp
	Msi2-iso-R	GTAGAGATCGGCGACAGGTCC	
Msi2 F3/R3	Msi2-iso2-F1	TCGAGGATCAGGCTCCAACCC	175 bp
	Msi2-iso2-R1	CAAAGGTCCAGCTATGCCGTG	
Msi1	m-Msi1-F1	AGTTGGCAGACCACGCAGGAAG	131 bp
	m-Msi1-R1	TGGTCCATGAAAGTGACGAAGC	
Numb F1/R1	Numb-F1	ACTACGGCAAAGCTTCAGGAG	185 bp
	Numb-R1	ATCTCTTTACGGCATCTTCAC	
Numb F2/R2	Numb-F2	TCGGCGATGGATCTGTCATTG	185 bp
	Numb-R2	TGACACGGAATGATCCTTCTC	
Sox7	Sox7-F1	AGGGGACAAGAGTTCGGAA	186 bp
	Sox7-R1	GCCTCATCCACATAGGGTC	
Tfec	Tfec-F1	AAAAAGAACAACAGAGAGCCC	188 bp
	Tfec-R1	CTCAGGATGGGTCTGCTGC	

### Knockdown and overexpression of Msi2

Msi2 was knocked down in D3 ESC that had been seeded at a density of 10^5^ cells per well in a 6-well plate. One day later, cells were infected with lentiviruses that express either the scrambled shRNA or shRNAs targeting Msi2 (Msi2 shRNA #1, #4, or #5). The protocol for infection of D3 ESC with lentiviruses has been described previously [13]. Flag-tagged Msi2 isoform 1 and Flag-tagged Msi2 isoform 2 were individually expressed from inducible transgenes stably integrated into i-Msi2.1-D3 ESC and i-Msi2.2-D3 ESC, respectively. The recombinant proteins were induced by the addition of 1 µg/ml Dox.

### AP staining and western blot analysis

Protocols for alkaline phosphatase (AP) staining, preparation of nuclear extracts and western blot analysis have been described previously [13]. Msi2 protein levels were determined with a Msi2 antibody (ab-76148, Abcam, Cambridge, MA, 1∶2,000). HDAC1 was used as the loading control and probed with an HDAC1 antibody (ab-7028, Abcam, 1∶5,000). Sox2 (#2683-1, Epitomics, Burlingame, CA, 1∶5,000) and Oct 4 (sc-8628, Santa Cruz, Santa Cruz, CA, 1∶500) antibodies were used to detect pluripotency markers. Msi2, HDAC1, and Sox2 primary antibodies were detected with an anti-rabbit-IgG-AP secondary antibody (A3687, Sigma-Aldrich,1∶10,000). Oct4 primary antibody was detected with an anti-goat-IgG-AP secondary antibody (A4187, Sigma-Alrich, 1∶10,000).

### Analysis of RNA expression

D3 ESC were infected with lentiviruses that express either scrambled shRNA or Msi2 shRNA #1 for 24 hours followed by selection with puromycin for 24 hours. Cells were subcultured 48 hours after puromycin selection at a low density (4,500 cells per cm^2^). Cells were maintained for 4 days in normal ES cell media followed by RNA isolation and cDNA synthesis as described previously [2]. Expression of ES-related genes and lineage-specific genes in D3 ESC treated with Msi2 shRNA #1 and scrambled shRNA were analyzed by SYBR Green (SuperArrayBioscience Corporation, Federick, MD) quantitative Real-Time polymerase chain reaction (RT-qPCR) [2]. Similarly, i-Msi2.1-D3 and i-Msi2.2-D3 were treated with or without 1 µg/ml Dox for 48 hours prior to plating at 4,500 cells/cm^2^. Cells were then treated with and without Dox in the presence of 5 µM RA for 48 hours, followed by the removal of LIF for 48 hours to further differentiate the cells prior to the RNA extraction and cDNA synthesis. Primers for Msi2 isoforms, Msi1, Numb, Sox7, and Tfec are provided in Table 3. Primers for ES cell- and lineage-specific transcripts have been described previously [2], [3], [17].

RNA isolated from D3 ESC infected with lentiviruses that express either scrambled shRNA or Msi2 shRNA #1, as described above, was used for genome-wide RNA expression analysis. Sense-strand cDNA was generated from 300 ng total RNA using the Ambion WT Expression kit for Affymetrix Whole Transcript Expression Arrays (Affymetrix, Santa Clara, CA). This cDNA was fragmented and labeled using the GeneChip® WT Terminal Labeling and Hybridization (Affymetrix) followed by hybridization for 16 hours at 45°C to an Affymetrix GeneChip Mouse Gene 1.0 ST (Affymetrix). Gene chips were washed and stained with the Affymetrix Fluidics Station 450 (Affymetrix) prior to being scanned by the Affymetrix GeneChip Scanner 3000 7G (Affymetrix). Data was analyzed with Affymetrix Expression Console software (Affymetrix) using Robust Multichip Analysis (RMA) for normalization. Data collection and analysis were performed by the University of Nebraska Medical Center DNA Microarray Core Facility. Microarray data was sorted for genes that increase or decrease 2-fold or more when Msi2 shRNA #1 was compared to the control scrambled shRNA. Of these genes, the Database for Annotation, Visualization and Integrated Discovery (DAVID) was used to classify them into broad-based cellular and molecular functions. [18], [19]. All microarray data is available on Gene Expression Omnibus (Accession No. GSE33882, GEO, http://www.ncbi.nlm.nih.gov/geo/).

### Cloning efficiency

Cells were plated at clonal densities and maintained in ES-cell media for up to 6 days, at which point the number of colonies that exhibited only the morphology of ESC, a mix of ESC and differentiated cells, or only cells with a differentiated morphology were counted in 10 random 40× fields by an observer unaware of sample designation.

## Supporting Information

Figure S1 **Knockdown of Msi2 results in minimal changes to pluripotency markers.** The D3 ESC were infected with lentiviruses that express scrambled (Scr) shRNA, shRNA #1, shRNA #4, or shRNA #5 sequences. Two days after infection, the cells were subjected to puromycin selection for 24 hours. After selection, the cells were subcultured and grown for an additional 24 hours before nuclear extracts were harvested for western blot analysis of pluripotency markers. HDAC1 was used as the loading control for quantification.
(TIF)

Figure S2 **Knockdown of Msi2 results in global changes in gene expression.** (**A**) Seven days post-infection, RNA was isolated from the D3 ESC infected with lentiviruses that express either the scrambled (Scr) shRNA sequence or the Msi2 shRNA#1 sequence. RNA was used for microarray analysis as described in the Materials and Methods. The dotted lines represent a 2-fold increase (bottom) or 2-fold decrease (top) in RNA expression relative to RNA isolated from the D3 ESC infected with the scrambled shRNA control sequence. (**B**) Gene ontology analysis of genes whose expression changes when Msi2 is knocked down. Gene ontology analysis was conducted for the genes whose expression increased (left side) or decreased (right side) two-fold or more in the Msi2 knockdown cell population. For this analysis, we used the Database for Annotation, Visualization and Integrated Discovery (DAVID).
(TIF)

Table S1 **Genes more highly expressed (>1.9-fold, <2.5-fold) in Msi2-shRNA #1 treated ESC.**
(XLS)

Table S2 **Genes more highly expressed (>1.9-fold, <2.5-fold) in control scr-shRNA treated ESC.**
(XLS)

Table S3 **Gene ontology heatmap of transcripts induced >2-fold in Msi2-shRNA#1 treated ESC.**
(XLS)

Table S4 **Gene ontology heatmap of transcripts induced >2-fold in scr-shRNA treated ESC.**
(XLS)
