# Supplementary figures and images for: Musashi2 Is Required for the Self-Renewal and Pluripotency of Embryonic Stem Cells
Source: PLoS One. 2012 Apr 4;7(4):e34827. doi: 10.1371/journal.pone.0034827 (PMC3319613; doi:10.1371/journal.pone.0034827)

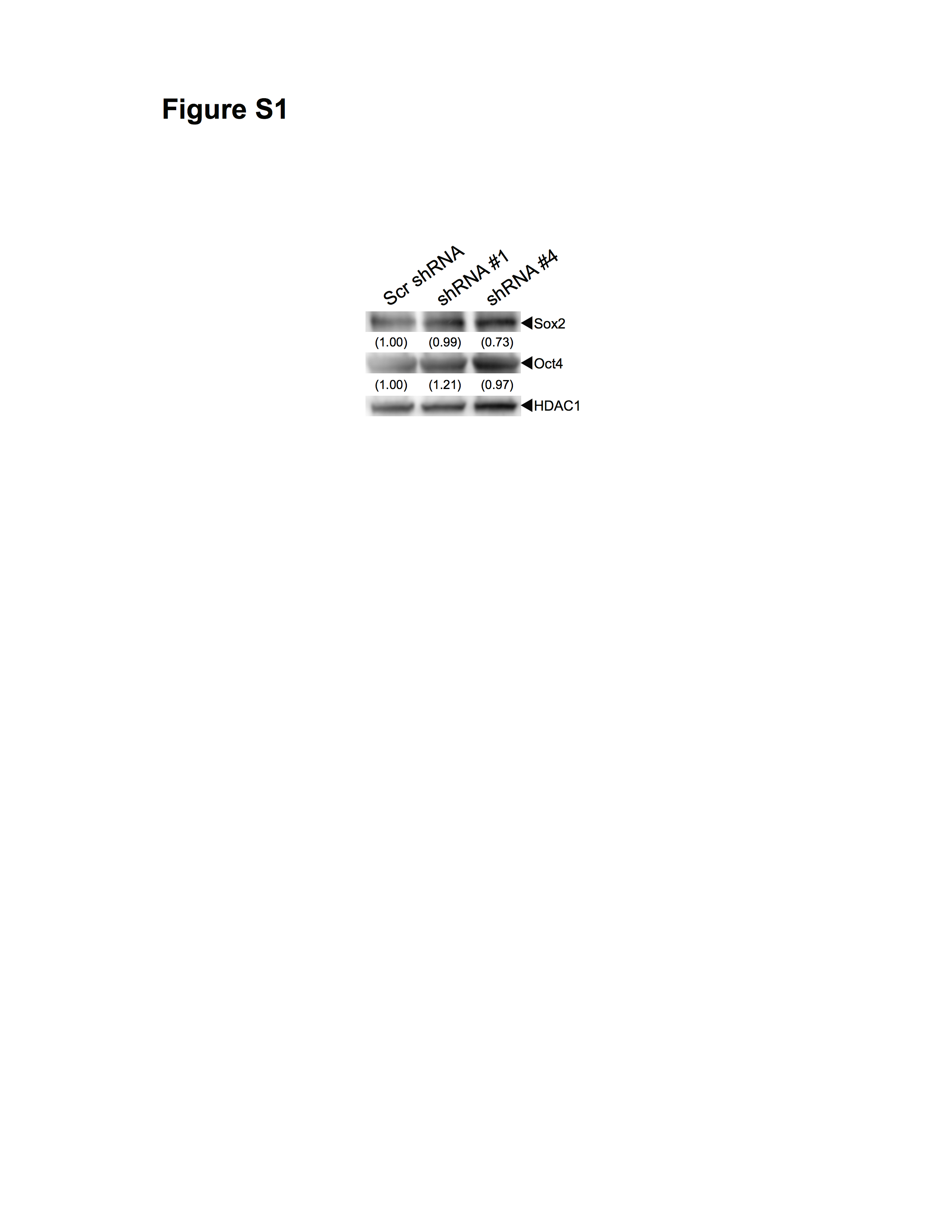

Supplement: Figure S1 — Knockdown of Msi2 results in minimal changes to pluripotency markers. The D3 ESC were infected with lentiviruses that express scrambled (Scr) shRNA, shRNA #1, shRNA #4, or shRNA #5 sequences. Two days after infection, the cells were subjected to puromycin selection for 24 hours. After selection, the cells were subcultured and grown for an additional 24 hours before nuclear extracts were harvested for western blot analysis of pluripotency markers. HDAC1 was used as the loading control for quantification. (TIF) [file pone.0034827.s001.tif]

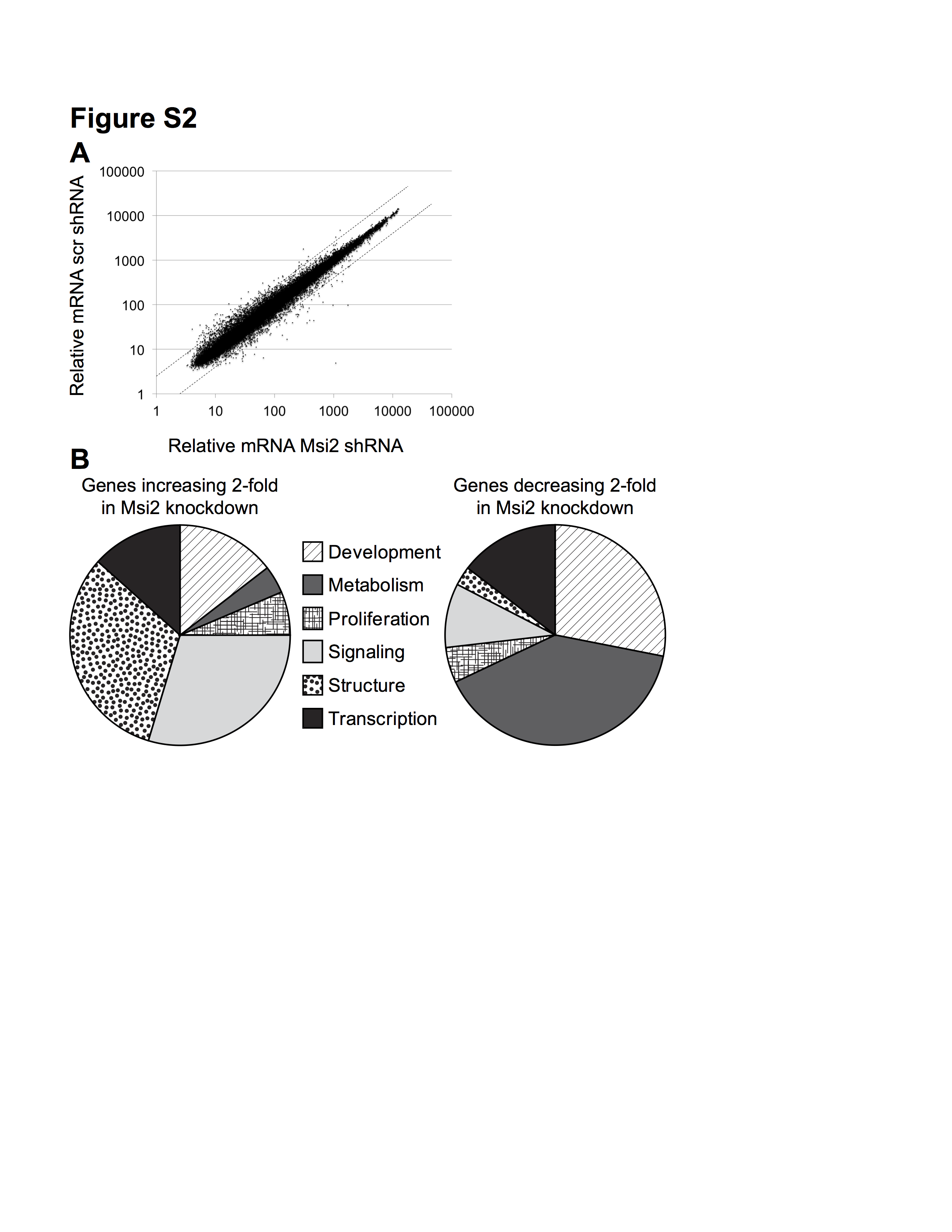

Supplement: Figure S2 — Knockdown of Msi2 results in global changes in gene expression. (A) Seven days post-infection, RNA was isolated from the D3 ESC infected with lentiviruses that express either the scrambled (Scr) shRNA sequence or the Msi2 shRNA#1 sequence. RNA was used for microarray analysis as described in the Materials and Methods. The dotted lines represent a 2-fold increase (bottom) or 2-fold decrease (top) in RNA expression relative to RNA isolated from the D3 ESC infected with the scrambled shRNA control sequence. (B) Gene ontology analysis of genes whose expression changes when Msi2 is knocked down. Gene ontology analysis was conducted for the genes whose expression increased (left side) or decreased (right side) two-fold or more in the Msi2 knockdown cell population. For this analysis, we used the Database for Annotation, Visualization and Integrated Discovery (DAVID). (TIF) [file pone.0034827.s002.tif]
